# Artificial Intelligence in Perioperative and Pain Care: Knowledge, Attitudes, and Adoption Barriers Among Anesthesiologists

**DOI:** 10.7759/cureus.113479

**Published:** 2026-07-27

**Authors:** Charmi Bhimbha, Abhilasha Motghare, Palak Ahir, Parth Jani, Mohit Suva, Niyati N Pandya

**Affiliations:** 1 Anaesthesiology, All India Institute of Medical Sciences, Rajkot, Rajkot, IND; 2 Anaesthesiology, Pain Medicine and Critical Care, All India Institute of Medical Sciences, Rajkot, Rajkot, IND; 3 Internal Medicine, All India Institute of Medical Sciences, Rajkot, Rajkot, IND; 4 Psychiatry and Behavioural Sciences, All India Institute of Medical Sciences, Rajkot, Rajkot, IND

**Keywords:** adoption barriers, artificial intelligence in anesthesia, attending anesthesiologists, awareness for artificial intelligence, knowledge attitude, perioperative care

## Abstract

Background

The integration of artificial intelligence (AI) in anesthesiology holds immense potential to revolutionize perioperative care. Despite the growing interest in AI, limited research exists on anesthesiologists’ knowledge, attitudes, and practical exposure to AI-based tools. This study aimed to assess the knowledge, attitudes, and awareness of AI applications in perioperative care of anesthesiologists with AI applications in perioperative care, spanning preoperative, intraoperative, and postoperative domains.

Methodology

A structured questionnaire was distributed among anesthesiologists across six major cities of Gujarat, India: Ahmedabad, Vadodara, Surat, Rajkot, Jamnagar, and Bhavnagar. The survey included demographic details, awareness of AI applications, attitudes toward AI adoption, and perceived challenges. Descriptive and inferential statistics were used to analyze the data.

Results

Among 385 respondents, 94.0% (n = 362) had heard of AI in healthcare, but only 63.6% (n = 245) were aware of its role in anesthesia. Knowledge assessment categorized 21.3% (n = 82) as having good knowledge, 70.9% (n = 273) as average, and 7.8% (n = 30) as poor. While 90.4% (n = 348) agreed that AI training could enhance adoption, 84.4% (n = 325) believed AI would ease their workload. However, 9.6% (n = 37) were concerned that AI-equipped doctors might replace those without AI expertise. The most cited barriers to AI adoption were lack of knowledge (84.4%, n = 325), legal concerns (27.3%, n = 105), and limited validation studies (16.9%, n = 65). Despite these challenges, 94.0% (n = 362) expressed willingness to read AI literature, and 93.2% (n = 359) reported improved knowledge post-survey. A statistically significant association was observed between years of work experience and AI knowledge levels (p < 0.01).

Conclusions

This study highlights a growing interest in AI among anesthesiologists but also underscores substantial knowledge gaps and implementation challenges. While attitudes toward AI are largely positive, concerns regarding training, validation, and medico-legal implications must be addressed. Strengthening AI education, fostering interdisciplinary collaboration, and ensuring ethical integration into clinical practice will be crucial for optimizing AI’s role in anesthesia. Future research should focus on validating AI tools and assessing their clinical impact.

## Introduction

The practice of anesthesiology is being reshaped by continuous technological innovation. Artificial intelligence (AI), the capacity of computational systems to mimic human cognitive functions [1,2], has emerged as a transformative influence in perioperative medicine. By augmenting clinical decision-making, streamlining repetitive tasks, and enhancing patient outcomes, AI is fundamentally shifting healthcare paradigms [2-4]. Within anesthesiology, emerging AI applications promise refined preoperative risk stratification, heightened intraoperative vigilance, and accelerated postoperative recovery, thereby advancing the goals of precision medicine [5,6]. Concrete examples include closed-loop anesthesia delivery systems and machine learning algorithms that predict the bispectral index from raw electroencephalography signals, both of which improve hemodynamic stability and the accuracy of depth-of-anesthesia monitoring [5,7]. In regional anesthesia, AI-enhanced ultrasound guidance has already demonstrated increased needle placement accuracy and procedural success rates [8].

Despite these encouraging developments, the existing literature has centered predominantly on technical feasibility and proof-of-concept studies, paying comparatively little attention to the viewpoints of frontline anesthesiologists. To date, research has not systematically evaluated clinicians’ familiarity with AI across all perioperative phases, nor has it quantified the perceived barriers to adoption, such as fundamental knowledge gaps, concerns about algorithmic interpretability, medicolegal ambiguity, or the demand for rigorous external validation [9,10]. Consequently, the readiness of anesthesiologists to incorporate AI into routine clinical workflow remains largely unknown.

Despite growing interest in artificial intelligence in perioperative care, there is limited data on anesthesiologists’ awareness, attitudes, and readiness for AI adoption in the Indian context, particularly in Gujarat. Therefore, in the present study, the primary objective of this study was to assess anesthesiologists’ knowledge and attitudes regarding AI applications in perioperative care. Secondary objectives were to identify barriers and facilitators to AI adoption and to evaluate factors associated with AI awareness and acceptance among anesthesiologists practicing in Gujarat, India. The paper begins with a concise overview of AI applications relevant to anesthesiology, describes the survey methodology used to capture clinician perspectives, presents the findings, discusses their implications for education and policy, and concludes with actionable recommendations for the safe and effective integration of AI in the era of digital transformation [10].

## Materials and methods

All methods described below are directly relevant to the study objectives of evaluating anesthesiologists’ knowledge, attitudes, barriers, and facilitators regarding AI integration. A cross-sectional online survey was employed among anesthesiologists practicing in six major cities of Gujarat (Ahmedabad, Vadodara, Surat, Rajkot, Jamnagar, and Bhavnagar), selected for their established medical infrastructure and diverse clinical settings. A structured, self-administered questionnaire was distributed to postgraduate residents, senior residents, and consultants who were members of the Indian Society of Anesthesiologists (ISA) and provided consent. The questionnaire was developed based on a review of relevant literature and was assessed for content validity by a panel of five senior consultants in anesthesiology. Each item was rated on a five-point Likert scale for relevance, clarity, and appropriateness (1 = poor, 5 = excellent), with higher scores indicating better suitability. Items receiving consistently high ratings were retained, while those with lower ratings were revised based on expert feedback to improve clarity and relevance. Exclusion criteria included non‑anesthesiologists, practitioners outside Gujarat, and incomplete responses. The questionnaire that was distributed has been included in the Appendices section.

Study design

This was a cross‑sectional, questionnaire‑based study without a control or randomization. The final study design was approved by the Research Cell at All India Institute of Medical Sciences, Rajkot. Convenience sampling was used for recruitment. Based on ISA records of approximately 1,600 anesthesiologists in the selected cities, the required sample size was calculated using the formula N = Z² × P × (1‑P) / E², where Z = 1.96 (95% confidence level), P = 0.5 (maximum variability), and E = 0.05 (5% margin of error), yielding a target of 385 participants to ensure adequate statistical power. Participants were recruited through professional networks and institutional contacts using an online survey platform, and participation was voluntary. The survey was hosted on a secure online platform; reminder messages were sent at predefined intervals to non‑respondents. The survey was designed using a Google Forms platform with mandatory response fields, ensuring that all submitted questionnaires were complete; therefore, no missing data were present for analysis. Automated validation checks ensured response completeness, duplicate submissions were excluded, and data were anonymized before analysis.

Statistical methods

Quantitative data were analyzed using descriptive statistics (percentages) to summarize participant demographics and responses across knowledge (questions 4‑15), barriers (Q16), facilitators (Q17, Q18, Q20), and sensitization (Q21, Q22). Attitudes toward AI (Q17‑22) were analyzed qualitatively to assess prevailing perspectives. For knowledge scoring, responses to Q4‑15 were scored as correct (1), partially correct (0.5), incorrect (0); each participant’s total knowledge score was calculated. Based on percentile distribution, participants were categorized as good knowledge (above 75th percentile), average knowledge (25th‑75th percentile), and poor knowledge (below 25th percentile). Attitude assessment (Q17‑22) categorized responses as positive or negative based on agreement with AI adoption, training, and investment. Barriers (Q16) and facilitators (Q17, Q18, Q20) were analyzed by frequency of selected responses. Sensitisation (Q21, Q22) was assessed through willingness to engage with AI literature and self‑reported knowledge improvement post‑questionnaire. Data were analyzed using SPSS software (IBM Corp., Armonk, NY, USA).

Ethical considerations

Ethical approval was obtained from the Institutional Ethics Committee of AIIMS Rajkot (IEC approval number: AIIMS/RAJKOT/5th IEC/FB/46 dated 5/8/2024). The study was conducted in accordance with the Declaration of Helsinki. Electronic informed consent was obtained from all participants before accessing the questionnaire; the consent form detailed the study objectives, voluntary participation, and confidentiality measures. No personally identifiable information was collected. This study received no external funding, and the authors declare no conflicts of interest.

## Results

Participant demographics

**Figure 1:**
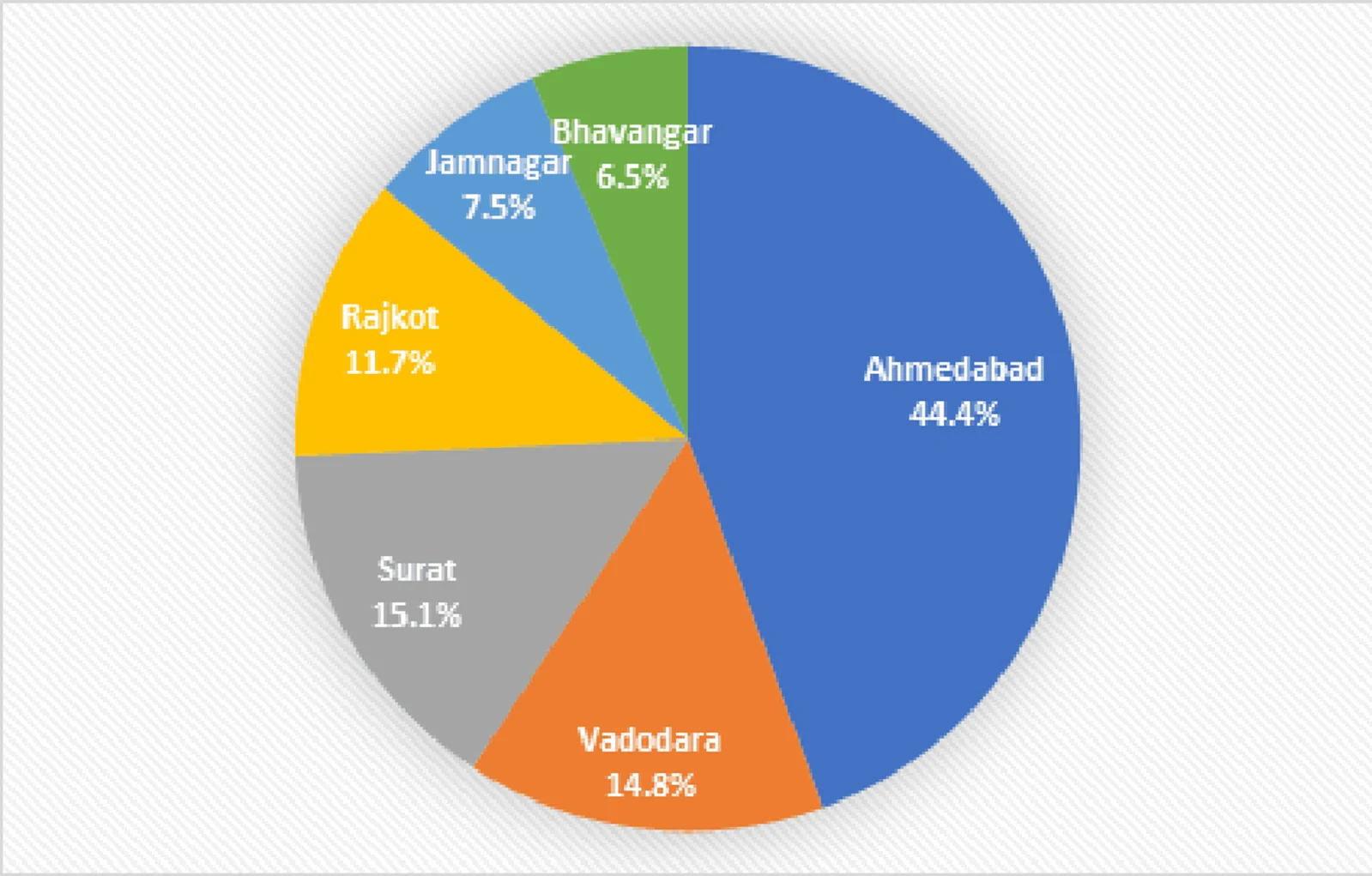
Distribution of participants in various cities of Gujarat. Ahmedabad 44.4% (n = 171), Surat 15.1% (n = 58), Vadodara 14.8% (n = 57), Rajkot 11.7% (n = 45), Jamnagar 7.5% (n = 29), and Bhavnagar 6.5% (n = 25).

A total of 385 anesthesiologists participated, representing a response rate of 48.1% (n = 385 out of 800). The response rate was calculated based on the 800 anesthesiologists who received the questionnaire after filtering out incorrect contact details from an initial pool of 1,600 anesthesiologists across six major cities in Gujarat. Participants were distributed across Ahmedabad 44.4% (n = 171), Surat 15.1% (n = 58), Vadodara 14.8% (n = 57), Rajkot 11.7% (n = 45), Jamnagar 7.5% (n = 29), and Bhavnagar 6.5% (n = 25) (Figure 1).

**Figure 2:**
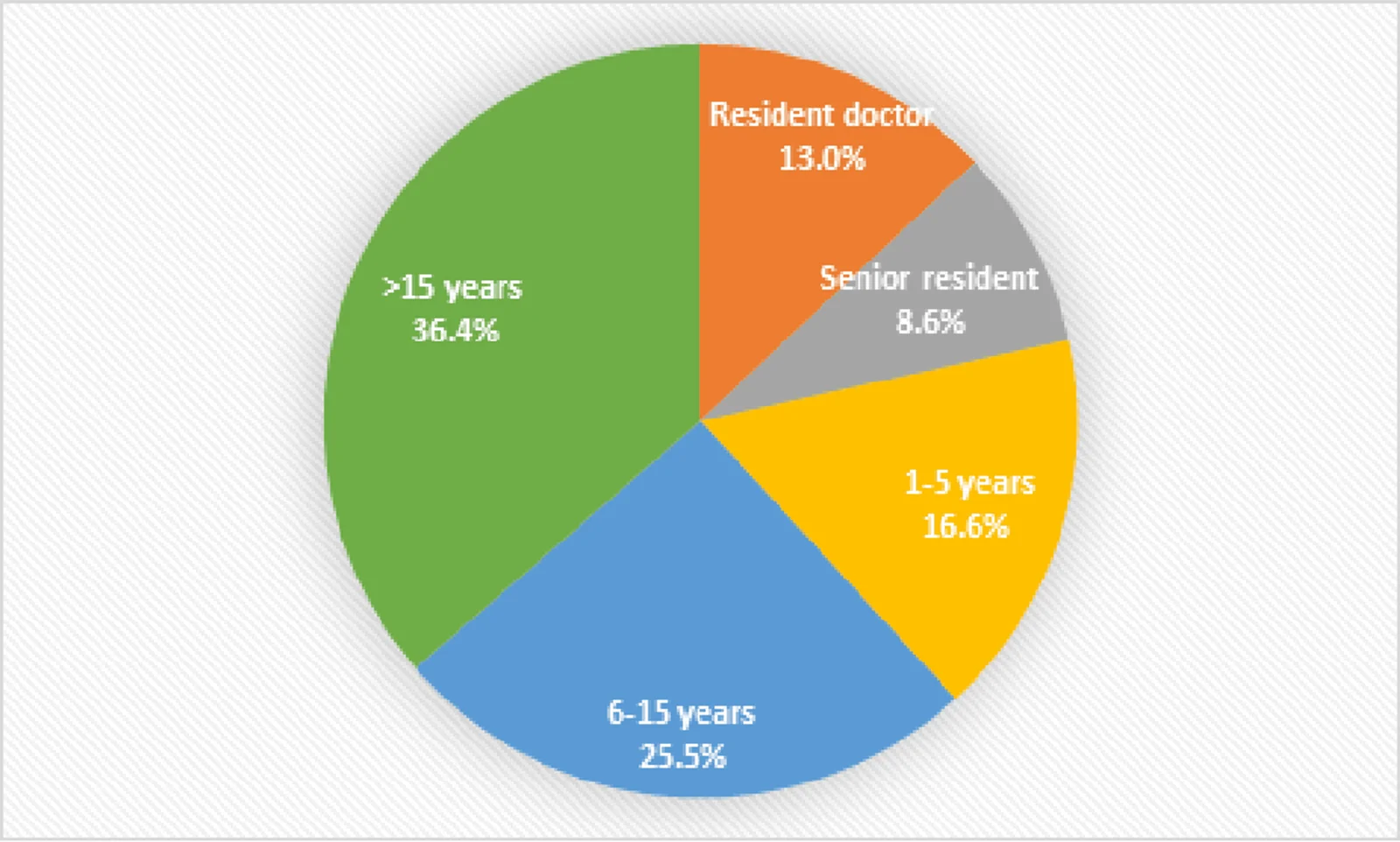
Distribution according to work experience. Resident doctors 13% (n = 50), Senior residents 8.6% (n = 33), 1-5 years of experience 16.6% (n = 64), 6-15 years of experience 25.5% (n = 98) and >15 years of experience 36.4% (n = 140).

The distribution of work experience was as follows: resident doctors 13.0% (n = 50), senior residents 8.6% (n = 33), 1‑5 years of experience 16.6% (n = 64), 6‑15 years of experience 25.5% (n = 98), and >15 years of experience 36.4% (n = 140). The mean age of participants was 38 years (range = 27-70) (Figure 2).

Knowledge of artificial intelligence in anesthesia

Assessment of knowledge was based on responses to 11 questions exploring familiarity with AI applications, exposure to AI literature, and awareness of AI in anesthesia practice. Awareness of AI in healthcare was reported by 94.0% (n = 362), while 6.0% (n = 23) had not heard of it. Awareness of AI’s role in anesthesia practice was present in 63.6% (n = 245), whereas 36.4% (n = 140) were not aware. Exposure to AI literature was reported by 24.2% (n = 93); 75.8% (n = 292) had not read any articles on AI. Among those who had read AI‑related articles, 44.3% (n = 58) found the content difficult to understand, while 55.7% (n = 73) did not. The most common challenges cited in understanding AI literature were understanding technical terminologies (33.3%, n = 31), evaluating clinical usefulness (60.9%, n = 57), interpreting results (18.4%, n = 17), and understanding methods (29.9%, n = 28).

Key areas where participants demonstrated AI knowledge included preoperative AI applications (risk stratification 76.6% (n = 295); airway evaluation 40.8% (n = 157); physical status assessment 26.8% (n=103), intraoperative AI applications (monitoring depth of anesthesia 76.9% (n=296); predicting adverse events 21.5% (n=83); ultrasound anatomical structure detection 17.4% (n = 67)), and postoperative AI applications (managing postoperative pain 75.6% (n = 291); predicting adverse events 24.7% (n = 95); and predicting rebound pain 15.8% (n = 61)).

**Figure 3:**
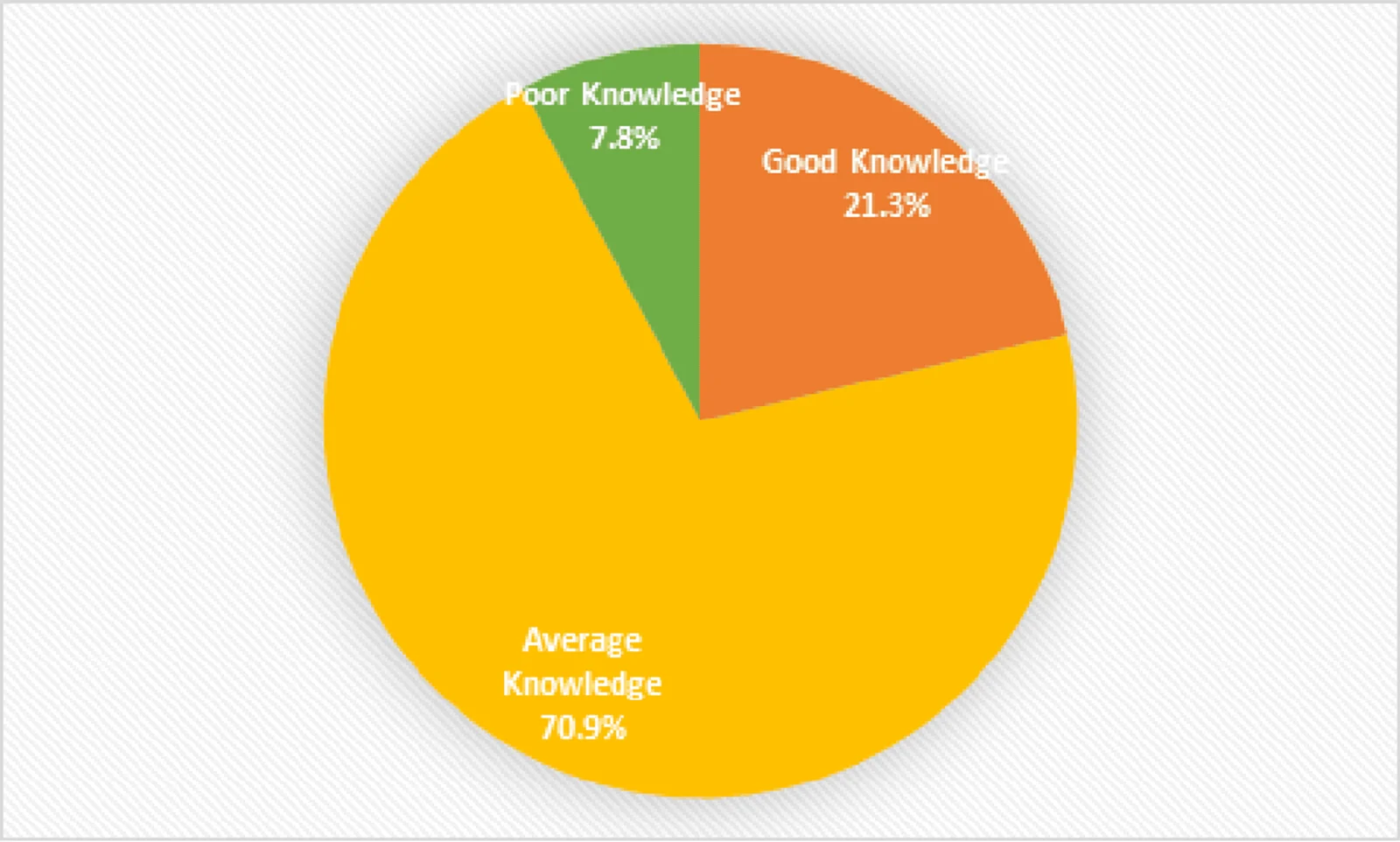
Distribution of knowledge scores. Good knowledge: scores above 6 21.3% (n = 82), average Knowledge: scores between 3 and 6 70.9% (n = 273), and poor knowledge: scores below 3 7.8% (n = 30).

Based on total knowledge scores, participants were categorized into the following three groups: good knowledge (scores above 6, top 25%) comprised 21.3% (n = 82); average knowledge (scores between 3 and 6, middle 50%) comprised 70.9% (n = 273); poor knowledge (scores below 3, bottom 25%) comprised 7.8% (n = 30) (Figure 3).

Attitudes toward artificial intelligence integration

Regarding training in AI, 90.4% (n = 348) agreed that dedicated AI training would encourage adoption in anesthesia practice. For investment in AI, 52.5% (n = 202) moderately supported AI investment, 35.6% (n = 137) strongly advocated for it, and 3.1% (n = 12) opposed AI investment. Concerning the future of AI in anesthesia, 84.4% (n = 325) believed AI would make their work easier; 9.6% (n = 37) anticipated that AI‑trained doctors might replace those without AI expertise, and 2.9% (n = 11) expressed concerns about AI replacing anesthesiologists entirely. Perceived benefits of AI included reducing workload (50.9%, n = 196), improving patient care (48.6%, n = 187), and decreasing hospital stays (42.3%, n = 163). Willingness to engage with AI literature post‑study was expressed by 94.0% (n = 362) of participants. Perceived knowledge improvement (enhanced awareness of AI applications) was reported by 93.2% (n = 359).

Barriers to artificial intelligence adoption

The most commonly identified barriers were lack of knowledge or training on AI (84.4%, n = 325), concerns about reliability and potential errors in AI systems (19.0%, n = 73), limited validation studies available (16.9%, n = 65), and legal and medical liability concerns (27.3%, n = 105).

Facilitators for artificial intelligence adoption

Participants highlighted structured training programs (90.4%, n = 348), evidence demonstrating the effectiveness and safety of AI systems, and collaborative efforts between anesthesiologists, engineers, and policymakers as facilitators that could promote AI adoption.

Sensitization outcomes

Post‑study analysis indicated that 94.0% (n = 362) of participants expressed interest in reading AI‑related literature, and 93.2% (n = 359) reported an improved understanding of AI applications following the questionnaire.

## Discussion

This investigation evaluated the knowledge, attitudes, barriers, and facilitators related to AI adoption among 385 anesthesiologists practicing in six major cities of Gujarat. The principal findings were as follows: 94% of respondents had heard of AI in healthcare, yet only 63.6% were aware of its specific applications within anesthesia. Knowledge scores revealed that 21.3% of participants possessed good knowledge, 70.9% had average knowledge, and 7.8% demonstrated poor knowledge. Attitudes toward AI were predominantly favorable, with 90.4% supporting dedicated AI training and 84.4% believing that AI would ultimately ease their clinical workload. The barrier cited most frequently was a lack of knowledge or training (84.4%), followed by legal concerns (27.3%), doubts about reliability (19%), and a perceived scarcity of robust validation studies (16.9%). Key facilitators included structured training programs (90.4%), demonstrable evidence of effectiveness, and interdisciplinary collaboration. Notably, after completing the study questionnaire, 94% of participants expressed a willingness to engage with AI literature, and 93.2% reported an improved awareness of the topic.

The observation that only 63.6% of participants were aware of AI applications in anesthesia, despite 94% having heard of AI in healthcare generally, exposes a distinct knowledge gap within the specialty. This finding is consistent with the work of Estrada Alamo et al. [11] and aligns with broader evidence that clinicians frequently lack a deep technical understanding of AI even when they possess general awareness of its existence [6]. Moreover, 60.9% of our participants reported difficulty in evaluating the clinical usefulness of AI tools, a challenge that directly mirrors documented struggles clinicians face when interpreting the machine learning literature [12]. The overall distribution of knowledge scores, a majority demonstrating only moderate familiarity, echoes patterns previously reported among other healthcare professional groups [13].

The strongly positive attitude toward AI training (90.4%) and the widespread belief that AI would make clinical work easier (84.4%) reflect the paradigm of “augmented intelligence,” wherein AI is seen as a tool that complements rather than replaces the clinician [4]. This optimism is consistent with earlier observations by Lonsdale et al., who noted similar expectations regarding AI’s potential to reduce cognitive load [9]. Nevertheless, a minority of participants harbored concerns about professional displacement: 9.6% worried that AI-trained physicians might supplant those without such expertise, and 2.9% feared the outright replacement of anesthesiologists. These figures are comparable to those reported by Estrada Alamo et al., where approximately 10% of anesthesiologist respondents expressed job displacement anxiety [11]. Such apprehensions, while not unfounded, can be effectively addressed through inclusive educational initiatives and a clear delineation of the clinician’s enduring role.

The predominant barrier, a lack of knowledge or training (84.4%), is a constraint repeatedly identified in the literature [7,8]. The secondary barriers of legal concerns (27.3%), reliability doubts (19%), and limited validation (16.9%) are equally well documented. For instance, Xu et al. demonstrated in a randomized controlled trial that an AI system improved anesthesia quality control during gastrointestinal endoscopy, yet they emphasized that validation across diverse clinical settings remains a prerequisite for widespread deployment [14]. The fact that 16.9% of our participants explicitly called for such validation evidence directly reinforces this principle. Regarding facilitators, the 90.4% endorsement rate for structured training programs exceeds the 72% reported in a comparable survey of perioperative risk prediction algorithms by Bihorac et al. [14], possibly reflecting a growing contemporary awareness of AI’s practical utility. Additionally, 50.9% of participants believed AI could reduce workload, and 48.6% anticipated improvements in patient care-perceived advantages that align with the demonstrated benefits of AI-assisted risk stratification tools such as the MySurgeryRisk algorithm [15].

The high post-study willingness to read AI literature (94%) and the self-reported improvement in knowledge (93.2%) suggest that even a brief, structured exposure can substantially enhance AI literacy, an insight with important ramifications for the design of future educational interventions. This may also reflect a survey-as-intervention effect, suggesting that engagement with the questionnaire itself could have influenced participants’ perceptions.

New contributions of this study

This work offers several novel contributions. It provides one of the very few region-specific (Gujarat, India) quantitative assessments of anesthesiologists’ AI readiness that spans all perioperative phases. It quantifies not only knowledge deficits but also the facilitators that can inform targeted interventions. The high post-study self-reported awareness gain (93.2%) suggests a possible “survey-as-intervention” effect, wherein engagement with AI-focused questions temporarily heightened perceived knowledge. However, as this measure is entirely subjective and was not compared with a baseline objective assessment, it should be interpreted with caution. Future studies could formally test this hypothesis using a pre-post knowledge design. Furthermore, unlike most prior studies focused on technical feasibility [7,9], this study directly captures frontline clinicians’ perspectives, including their specific difficulty in interpreting AI literature. Finally, the robust sample size (n = 385) and response rate (48.1%) provide a solid empirical foundation for future longitudinal comparisons. These strengths, together with the candidly discussed limitations, lay a foundation for more rigorous, longitudinal research.

Limitations

Several limitations must be acknowledged. Convenience sampling may restrict generalizability, as participants were recruited from six cities in Gujarat and may not represent anesthesiologists in other regions or countries. Self-reported data are susceptible to response bias, including a potential “survey-as-intervention” effect, wherein completing the questionnaire itself may have temporarily increased participants’ perceived AI awareness rather than reflecting sustained knowledge gains. The cross-sectional design precludes causal inference and the assessment of temporal changes. Additionally, although the questionnaire was assessed for content validity by senior consultants, no formal psychometric validation (e.g., reliability coefficients, exploratory factor analysis) was undertaken. The questionnaire measured familiarity and attitudes but not actual performance with AI tools. The use of a non-validated, albeit expert-reviewed, questionnaire could introduce measurement bias. Moreover, the “survey-as-intervention” effect described in the discussion is an inference based on self-reported perception and may not represent true, sustained learning. Future studies should employ larger, randomized samples, incorporate objective AI competency assessments, and utilize validated instruments.

Recommendations for practice

Structured AI training programs should be integrated into anesthesiology residency curricula and continuing medical education, with a focus on practical perioperative applications and the interpretation of AI-generated outputs. Healthcare institutions should invest in external validation studies before the clinical deployment of any AI system, directly addressing the reliability and validation concerns identified by 16.9% of respondents. Legal and regulatory frameworks must be established to clarify medicolegal liability when AI tools are employed in patient care, a need underscored by the 27.3% who cited legal apprehensions. Interdisciplinary collaboration among anesthesiologists, data scientists, and policymakers should be formalized to ensure that future AI tools are transparent, explainable, and seamlessly integrated into clinical workflows. Finally, given that 94% of participants expressed a readiness to engage with AI literature, professional societies and journals should curate accessible educational resources to nurture this interest and steadily elevate AI literacy across the specialty. Ultimately, the thoughtful integration of AI into anesthesiology aligns with the broader transition toward data-driven, high-performance medicine [4,16].

## Conclusions

This study reveals a clear duality among anesthesiologists: high general awareness of AI in healthcare but substantially lower awareness of its specific applications in anesthesia. The majority of participants demonstrated average to poor knowledge of AI in perioperative care, indicating a significant educational gap. Despite this, attitudes are strongly positive, with most supporting dedicated AI training and believing AI will ease their workload. A large majority expressed willingness to engage with AI literature after the study and reported improved awareness. The predominant barrier is lack of knowledge or training, followed by legal concerns, reliability concerns, and limited validation studies. Key facilitators identified include structured training programs, evidence of AI effectiveness, and interdisciplinary collaboration. These findings justify the conclusion that while enthusiasm for AI integration is high among anesthesiologists, targeted educational interventions, robust validation of AI tools, and clear medicolegal frameworks are essential prerequisites for safe and effective adoption. Without addressing these gaps, the potential of AI to enhance patient safety, reduce workload, and improve anesthetic care may be limited. Therefore, the take‑home message is that, as per the findings of the study, systematic integration of AI literacy into training and targeted strategies addressing the identified barriers are promising directions that merit further prospective evaluation.

## Appendices

Survey questionnaire

Study Title: Artificial Intelligence in Perioperative and Pain Care: Knowledge, Attitudes, and Barriers to Adoption among Anaesthesiologists

Instructions: Please answer all questions. For multiple-choice questions, select the one option that best applies unless instructed otherwise. For “check all that apply” questions, you may select more than one option. Follow skip patterns where indicated.

Section A: Demographics

Q1. Please mention your age. *

Q2. Years of work experience (in anaesthesia): *

Mark only one.

Resident doctor

Senior resident

1-5 years

6-15 years

>15 years

Q3. Please provide your Email ID: *

Q4. Please mention the city where you are currently practising anaesthesiology. *

Section B: General awareness and reading

Q5. Have you ever heard of the role of Artificial Intelligence (AI) in healthcare? *

Mark only one.

Yes

No

Q6. Are you aware of the role of AI in anaesthesia practice? *

Mark only one.

Yes

No

Q7. Have you ever read any article on AI? *

Mark only one.

Yes

No (If No, skip to Q10)

Q8. Did you find it difficult to understand? *

Mark only one.

Yes

No (If No, skip to Q10)

Q9. In which aspect did you find it most difficult? *

Mark only one. (After this question, proceed to Q10.)

Understanding of the methods

Understanding of the results

Understanding of terminologies used

Evaluate its clinical usefulness

Section C: Perioperative AI applications - awareness

Q10. Amongst the following anaesthesiology practices, where can AI‑based tools be applied? *

Check all that apply.

Preoperatively

Intraoperatively

Postoperatively

I don’t know

Q11. Amongst the following pre‑operative anaesthesiology practices, where can AI‑based tools be applied? *

Check all that apply.

Airways evaluation

Risk stratification

Physical status assessment

I don’t know

Q12. Which of the following pre‑operative implementations of AI have you heard of? *

Check all that apply.

AI model predicts a patient’s ASA PS on a continuous scale using the patient’s home medications and comorbidities.

AI model by using frontal facial images capable of identifying difficult to intubate patients, with performances superior to two conventional tests, Mallampati test and thyromental distance.

"MySurgeryRisk" score is able to predict the risk for 8 postoperative complications [acute kidney injury (AKI), sepsis, venous thromboembolism, intensive care unit (ICU) admission >48 h, mechanical ventilation >48 h, wound, neurologic, and cardiovascular complications].

No, I have never heard about any of the above applications of AI till now.

Q13. Amongst the following intra‑operative anaesthesiology practices, where can AI‑based tools be applied? *

Check all that apply.

Closed-loop anesthesia, monitoring the depth of anaesthesia

US anatomical structure detection

Managing intraoperative pain

Predicting adverse events, predicting hypoxemia, hypotension, predicting hypoxemia

Automating drugs administration

I don’t know

Q14. Which of the following intra‑operative implementations of AI have you heard of? *

Check all that apply.

Epidural space identification using a single-element Ultrasound transducer at the needle tip in 3D‑augmented environment.

AI algorithm in order to determine the needle insertion point using automated spinal landmark US imaging of the lumbar spine.

AI model (XGBoost) that predicts the grade of sedation required to successfully conduct a colonoscopy.

Medtronic & Minneapolis, models use the EEG signal to continuously predicts the bispectral index (BIS) for depth of anaesthesia monitoring.

No, I have never heard about any of the above applications of AI till now.

Q15. Amongst the following post‑operative anaesthesiology practices, where can AI‑based tools be applied? *

Check all that apply.

Managing postoperative pain

Predicting adverse events

Predicting rebound pain after peripheral nerve block

I don’t know

Q16. Which of the following post‑operative implementations of AI have you heard of? *

Check all that apply.

AI tools for pain risk prediction and supporting clinical decision making regarding acute pain service (APS).

Generalized additive model with neural networks (GAM‑NNs) capable of predicting mortality in patients undergoing general anesthesia.

AI tools giving predictive algorithm for detecting early signs of deterioration.

No, I have never heard about any of the above applications of AI till now.

Section D: Barriers, attitudes, and future perspectives

Q17. Which of these reasons would be / would be the main barriers to using an AI‑based tool? *

Check all that apply.

Not enough knowledge on AI in general

Lack of knowledge of the specific algorithm that leads to the result

Only few validation studies available on this issue

Legal and medical liability issues

Q18. Do you think that training dedicated to the use of new AI and Machine Learning (ML) technologies in the anesthesiology field can lead to greater use of the same? *

Mark only one.

Yes

No

Q19. Do you think that today it can be useful to invest in technologies based on AI and ML algorithms in anesthesia? *

Mark only one.

Much

Moderately

Little

Not at all

Q20. Which of the following do you agree with? *

Mark only one.

In the future you may get replaced by these new technologies.

In future your work will get easier with these technologies.

In future a doctor who knows AI and is AI equipped will replace a doctor who doesn’t know it.

In future these technologies will have no role in clinical practice.

Q21. Which of these advantages would be / would be the main one(s)? *

Check all that apply.

Decrease hospital stay by improving patient care

Improve quality of care

Decrease burden to over worked doctors

Implement monitoring systems

Section E: Post‑questionnaire self‑assessment

Q22. After filling this questionnaire, will you read any article/literature regarding AI? *

Mark only one.

Yes, I may read

I don’t have enough time post duty to read

No, I will not.

Q23. After filling the above questionnaire, has your knowledge on AI improved? *

Mark only one.

Yes, I came to know about few implementations of it

No, it’s same as earlier
